# Increased Renal Iron Accumulation in Hypertensive Nephropathy of Salt-Loaded Hypertensive Rats

**DOI:** 10.1371/journal.pone.0075906

**Published:** 2013-10-08

**Authors:** Yoshiro Naito, Hisashi Sawada, Makiko Oboshi, Aya Fujii, Shinichi Hirotani, Toshihiro Iwasaku, Yoshitaka Okuhara, Akiyo Eguchi, Daisuke Morisawa, Mitsumasa Ohyanagi, Takeshi Tsujino, Tohru Masuyama

**Affiliations:** 1 Cardiovascular Division, Department of Internal Medicine, Hyogo College of Medicine, Nishinomiya, Japan; 2 Division of Coronary Heart Disease, Department of Internal Medicine, Hyogo College of Medicine, Nishinomiya, Japan; 3 Department of Pharmacy, Hyogo University of Health Sciences, Kobe, Japan; Universidade Federal do Rio de Janeiro, Brazil

## Abstract

Although iron is reported to be associated with the pathogenesis of chronic kidney disease, it is unknown whether iron participates in the pathophysiology of nephrosclerosis. Here, we investigate whether iron is involved in the development of hypertensive nephropathy and the effects of iron restriction on nephrosclerosis in salt- loaded stroke-prone spontaneously hypertensive rats (SHRSP). SHRSP were given either a normal or high-salt diet for 8 weeks. Another subset of SHRSP were fed a high-salt with iron-restricted diet. SHRSP given a high-salt diet developed severe hypertension and nephrosclerosis. As a result, survival rate was decreased after 8 weeks diet. Importantly, massive iron accumulation and increased iron content were observed in the kidneys of salt-loaded SHRSP, along with increased superoxide production, urinary 8-Hydroxy-2′-deoxyguanosine excretion, and urinary iron excretion; however, these changes were markedly attenuated by iron restriction. Of interest, expression of cellular iron transport proteins, transferrin receptor 1 and divalent metal transporter 1, was increased in the tubules of salt-loaded SHRSP. Notably, iron restriction attenuated the development of severe hypertension and nephrosclerosis, thereby improving survival rate in salt-loaded SHRSP. Taken together, these results suggest a novel mechanism by which iron plays a role in the development of hypertensive nephropathy and establish the effects of iron restriction on salt-induced nephrosclerosis.

## Introduction

The number of patients with chronic kidney disease (CKD) is increasing, and CKD is associated with increased risk of sudden death [1]. Meanwhile, Hypertension is an important risk factor for CKD progression [2]. Since hypertensive nephropathy is commonly applied to CKD associated with essential hypertension, it is worthwhile to investigate the pathophysiology of hypertensive nephropathy.

Iron is a necessary element for life and plays an essential role in several metabolic processes. Nonetheless, excess iron accumulation accelerates the Fenton reaction, which leads to oxidative stress and cell damage. Interestingly, abnormal iron deposition is observed in the tubules of human chronic renal disease [3] and animal models of nephropathy [4]–[6]. In addition, we have recently reported that renal iron accumulation and expression of intracellular iron transport proteins, such as transferrin receptor 1 (TfR1) and divalent metal transporter 1 (DMT-1), are increased in the tubules of the well-established 5/6 nephrectomy rat model of CKD. Furthermore, we have shown that dietary iron restriction attenuates the development of renal damage in CKD model rats [7]. However, it is largely unknown whether iron and intracellular iron transport proteins participate in the pathophysiology of hypertensive nephropathy. In addition, the effects of iron restriction on the development of salt-induced nephrosclerosis remain unknown.

In the present study, we investigate whether iron is involved in the development of hypertensive nephropathy and the effects of iron restriction on nephrosclerosis in salt-loaded stroke-prone spontaneously hypertensive rats (SHRSP). Herein, we find that massive iron accumulation and increased iron content are observed in the kidneys of salt-loaded SHRSP and that iron restriction attenuates the development of hypertensive nephropathy in salt-loaded SHRSP.

## Materials and Methods

### Ethics Statement

All of our experimental procedures were approved by the Animal Research Committee of Hyogo College of Medicine (protocol #13-036), and were performed in accordance with the Guidelines on Animal Experimentation that were released by the Japanese Association for Laboratory Animal Science.

### Animal Models

7-week-old male SHRSP were purchased from SLC Japan and housed in the Hyogo College of Medicine Animal Care Facility. Rats were fed a normal diet (0.3% NaCl) for 1week. Afterwards, rats were randomly assigned to three groups and were given a normal salt diet (0.3% NaCl) ([Control] n = 6), a high-salt diet (8% NaCl) ([HS] n = 6), or a high-salt with iron-restricted diet ([HS+IR)] n = 6) for 4 weeks. The nutrients of a normal diet consist of cornstarch 33%, casein 22%, cellulose 5%, sucrose 30%, corn oil 5%, mineral mixture 4%, and vitamin mix 1%. Mineral mixture contains dicalcium phosphate dihydrate 0.43%, potassium dihydrogen phosphate 34.31%, sodium chloride 25.06%, ferric citrate 0.623%, magnesium sulfate 4.8764%, zinc chloride 0.02%, manganese (II) sulfate pentahydrate 0.121%, copper (II) sulfate pentahydrate 0.156%, potassium iodide 0.0005%, calcium carbonate 29.29%, ammonium molybdate tetrahydrate 0.0025%, and microcrystalline cellulose 5.11%. An iron-restricted diet was based on a normal diet, but with a mineral mixture free of ferric citrate [7]. Rats were maintained on a 12 hour light/dark cycle and had free access to food and water. Systolic blood pressure (SBP) and body weight were monitored every 1 week and behavior was assessed every day during the experiments. Urine samples were collected for 24 hours in metabolic cages after 3 weeks diet. After 4 weeks diet, rats were sacrificed under anesthesia with pentobarbital sodium (i.p.). The adequacy of anesthesia was confirmed by disappearance of eyelid reflex, corneal reflex, loss of muscular tone, and no response to surgical manipulation. Their blood was quickly withdrawn by abdominal aorta puncture, and serum was stored at −80°C before analysis. The tissues were excised and washed in phosphate-buffered saline. Afterwards, the tissues were quickly snap-frozen in liquid nitrogen and stored at −80°C. A part of each sample was fixed with buffered 4% paraformaldehyde. In a separate study, to examine the effects of iron restriction on survival rate, other 8-week-old SHRSP given Control (n = 9), HS (n = 12), and HS+IR (n = 9) were observed for 8 weeks diet.

### Assessments of Blood Pressure, Urine, Blood, and Renal Iron Content

SBP was measured by a noninvasive computerized tail-cuff system (MK-2000, Muromachi Kikai) [8]. Urinary concentrations of total protein and iron were determined by pyrogallol red method and atomic absorption method, respectively [7]. Urinary 8-Hydroxy-2′-deoxyguanosine (8-OHdG) levels were assessed by enzyme-linked immune sorbent assay (Japan Institute for the Control of Aging). Peripheral blood cell count, serum BUN, serum creatinine, and serum iron levels were determined as previously reported [8]. Renal iron content was determined by atomic absorption [9].

### RNA Extraction and Real-Time Quantitative RT-PCR

Total RNA was extracted from the kidney and liver using TRIzol reagent (Invitrogen) according to the manufacturer’s instructions. Total RNA was treated with DNase and reverse transcribed into cDNA using random primers (Applied Biosystems). Real-time PCR reactions were performed using the ABI PRISM 7900 with TaqMan Universal PCR Master Mix and TaqMan Gene Expression Assays (Applied Biosystems) [9]. The mRNA levels were normalized to GAPDH gene expression. TaqMan Gene Expression Assays were used as probes and primers for each gene as follows: heme oxygenase-1 (HO-1) (assay ID Rn01536933_m1), hepcidin (assay ID Rn00584987_m1), collagen type III (assay ID no. Rn01437683_m1), transforming growth factor-β (TGF-β) (assay ID Rn99999016_m1), CD68 (assay ID Rn01495634_g1), plasminogen activator inhibitor type 1 (PAI-1) (assay ID Rn01481341_m1), and glyceraldehyde-3-phosphatedehydrogenase (GAPDH) (assay ID no. Rn99999916_m1).

### Histomorphometric Analysis

Kidney tissues were fixed with buffered 4% paraformaldehyde, embedded in paraffin, and cut into 4-µm-thick sections. Periodic acid-Schiff and Masson’s trichrome staining were performed using serial sections. Ferric iron deposits were stained using berlin blue staining. Glomerular lesions and tubular lesions were evaluated by semiquantitative score using the method as previously described [10].

### Immunohistochemical Analysis

Renal sections were immunohistochemically stained with a primary mouse anti-CD68 antibody (AbD Serotec; dilution 1∶1000), a primary mouse anti-desmin antibody (Dako; dilution 1∶50), a primary mouse anti-TfR1 antibody (Zymed Laboratories; dilution 1∶200), a primary rabbit anti-DMT-1 with iron-responsive element antibody (Alpha Diagnostic; dilution 1∶100), and a primary rabbit anti-hepcidin-25 antibody (abcam; dilution 1∶1000). Immunostains were visualized with the use of an avidin-biotin-peroxidase conjugate and 3,3′-diaminobenzidine substrate. Every section was counterstained with hematoxylin. Quantification of CD68 positive cells and desmin staining was evaluated as previously described [7]. Terminal deoxynucleotidyl transferase–mediated dUTP nick-end labeling (TUNEL) staining was performed according to the manufacturer’s instruction (In situ apoptosis detection kit; TaKaRa). The section was counterstained with and DAPI. The number of TUNEL-positive cells was counted as previously described [7]. A part of kidney tissues was quickly embedded in Tissue-Tek OCT compound (Sakura Finetechnical Co) and snap-frozen in liquid nitrogen. Superoxide detection was performed as previously described [7].

### Transmission Electron Microscopy

For electron microscopy analysis, fresh kidney tissues were fixed with ice-cold buffer containing 4% paraformaldehyde, 5% glutaraldehyde, and 0.2 M Phosphate buffer (pH 7.4). The tissues were visualized as described previously [7].

### Statistical Analysis

Values are reported as the means ± SEM. Statistical analysis was performed using one way analysis of variance. Analysis of variance (Kruskal-Wallis test, followed by Mann-Whitney U test) was used for statistical comparisons. Kaplan-Meier analysis was used to assess survival rate. We considered that the differences were significant when the probability value was <0.05.

## Results

### Massive Iron Accumulation in the Renal Tubules of Salt-loaded SHRSP

**Figure 1:**
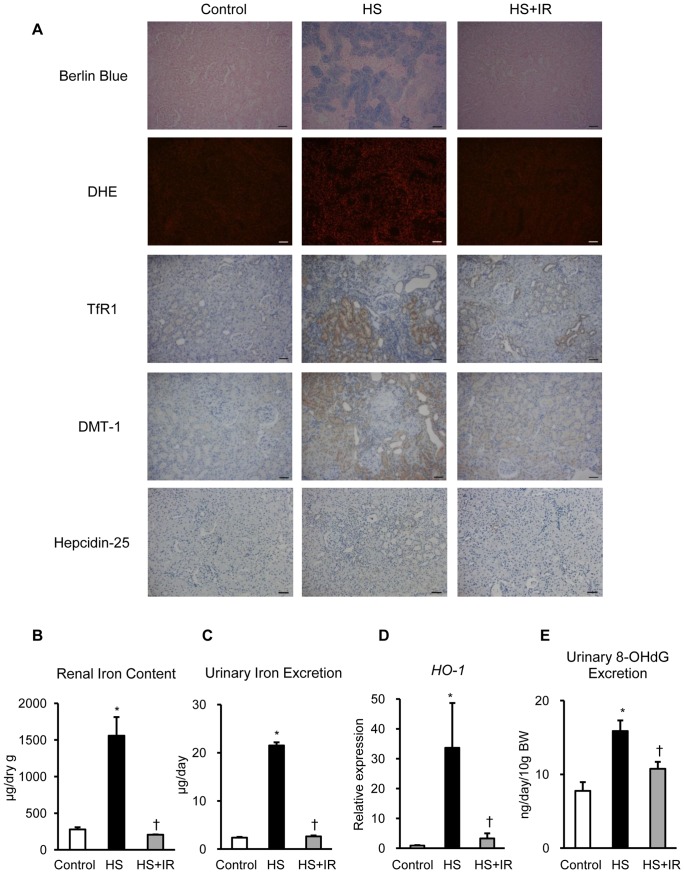
Massive Iron Accumulation in the Renal Tubules of Salt-Loaded SHRSP. (A) Representative images of berlin blue, DHE, TfR1, DMT-1, and hepcidin-25 staining of the kidney sections. Scale bars: 50 µm. (B) Renal iron content, (C) urinary iron excretion, (D) renal *HO-1* gene expression, and (E) urinary 8-OHdG excretion in the Control (white bar), HS (black bar), and HS+IR (gray bar) groups (n = 4–6 in each group). DHE, dihydroethidium; TfR1, transferrin receptor 1; DMT-1, divalent metal transporter 1; HO-1, heme oxygenase-1. Control, SHRSP fed a normal diet; HS, SHRSP fed a high-salt diet; HS+IR, SHRSP fed a high-salt with iron-restricted diet. *p<0.05 versus the Control group, ^†^p<0.05 versus the HS group.

HS diet increased SBP of SHRSP compared with a normal diet (Control, HS; 206.3±5.7, 270.0±11.1, mmHg, p<0.05). HS diet also increased urinary total protein excretion compared with a normal diet (Control, HS; 15.9±1.9, 198.9±23.6, mg/day, p<0.05). In order to investigate whether iron is involved in the development of hypertensive nephropathy in salt-loaded SHRSP, we assessed iron accumulation and intracellular iron transport proteins, such as TfR1 and DMT-1, in the kidney of these groups. Berlin blue staining revealed massive iron accumulation in the renal tubules of the HS group compared with the other groups (Figure 1A). In addition, DHE staining showed increased superoxide production in the kidney of the HS group (Figure 1A). Moreover, immunohistochemical analysis demonstrated that TfR1 and DMT-1 were strongly expressed in the tubules of both HS and HS+IR groups (Figure 1A). Hepcidin-25, the active form of iron-regulatory hormone hepcidin, was weakly expressed in the tubules of these groups, and hepcidin-25 positive cells tended to be increased in the HS group compared with the other groups (Figure 1A). Next, we evaluated renal iron content and urinary iron excretion in these groups. Of interest, renal iron content and urinary iron excretion were markedly increased in the HS group compared with the other groups (Figure 1B,C). Moreover, renal HO-1 gene expression and urinary 8-OHdG excretion were significantly increased in the HS group, whereas these increments were suppressed in the HS+IR group (Figure 1D,E). These data suggest that iron related oxidative damage may be associated with the pathophysiology of hypertensive nephropathy in salt-loaded SHRSP.

### Effects of Iron Restriction on Physiological Parameters and Proteinuria in Salt- loaded SHRSP

**Figure 2:**
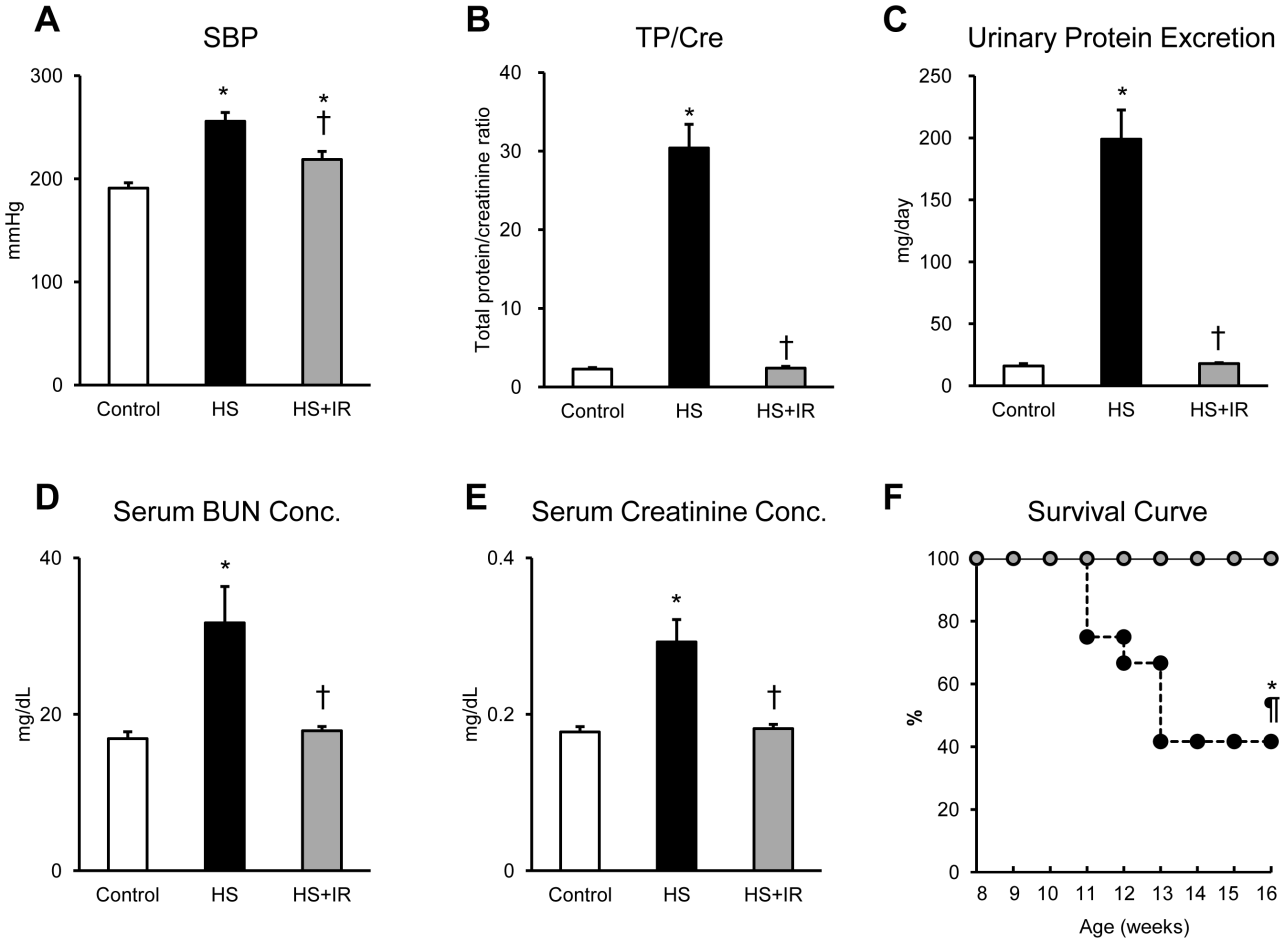
Effects of Dietary Iron Restriction on Systolic Blood Pressure, Proteinuria, and Prognosis in Salt-Loaded SHRSP. (A) SBP, (B) urinary total protein/creatinine ratio, (C) urinary total protein excretion, (D) serum BUN concentration, and (E) serum creatinine concentration in the Control (white bar), HS (black bar), and HS+IR (gray bar) groups (n = 6 in each group). (F) Survival rate after diet in the Control (white circle, n = 9), HS (black circle, n = 12), and HS+IR (gray circle, n = 9) groups. SBP, systolic blood pressure; TP/Cre, urinary total protein/creatinine ratio. *p<0.05 versus the Control group, ^†^p<0.05 versus the HS group, ^¶^p<0.05 versus the HS+IR group.

HS diet resulted in a increase in SBP, while the increment of SBP in the HS+IR group was smaller than in the HS group (Figure 2A), indicating that iron restriction in the presence of HS diet inhibited the increase in SBP. In addition, HS diet significantly increased proteinuria, serum BUN levels, and serum creatinine levels, whereas these increments were attenuated in the HS+IR group (Figure 2B–E). On the other hand, body weight was decreased in the HS group compared with the other groups. Blood hemoglobin, mean corpuscular volume, mean corpuscular hemoglobin, serum iron levels, and hepatic hepcidin gene expression were decreased in the HS+IR group (Table 1), indicating that hepatic hepcidin gene expression is down-regulated in response to iron deficiency in the HS+IR group. During the experimental period, some rats died of stroke suddenly (33%) in the HS group, and some rats were euthanized because they showed seizure, paralysis of hindlimb, and decreased activity (25%) in the HS group; however, none of rats died in the HS+IR group. Kaplan-Meier analysis showed that the HS+IR group had a better prognosis than the HS group (Figure 2F).

**Table 1:** Physiological parameters, hematologic parameters, and relative hepatic hepcidin gene expression levels in all groups at 12 weeks of age.

Parameters	Control	HS	HS+IR
Body weight (g)	268.1±4.9	198.7±0.7* †	272.3±17.7
Hemoglobin (g/dL)	16.3±0.2	15.5±0.6†	14.1±0.2*
MCV (µm^3^)	52.3±0.3	52.5±0.5†	46.5±0.3*
MCH (pg)	17.4±0.1	17.9±0.1†	15.5±0.2*
Serum iron levels (µg/dL)	231.6±26.6	248.3±56.0†	84±8.0*
Hepatic hepcidin gene expression	1.1±0.2	1.0±0.1†	0.004±0.002*

* p<0.05 versus Control group,

† p<0.05 versus HS+IR group. n = 6 per group. Control, SHRSP fed a normal salt diet; HS, SHRSP fed a high-salt diet; HS+IR, SHRSP fed a high-salt with iron-restricted diet; MCV, mean corpuscular volume; MCH, mean corpuscular hemoglobin.

### Effects of Iron Restriction on Renal Structure and Renal Gene Expression in Salt-loaded SHRSP

**Figure 3:**
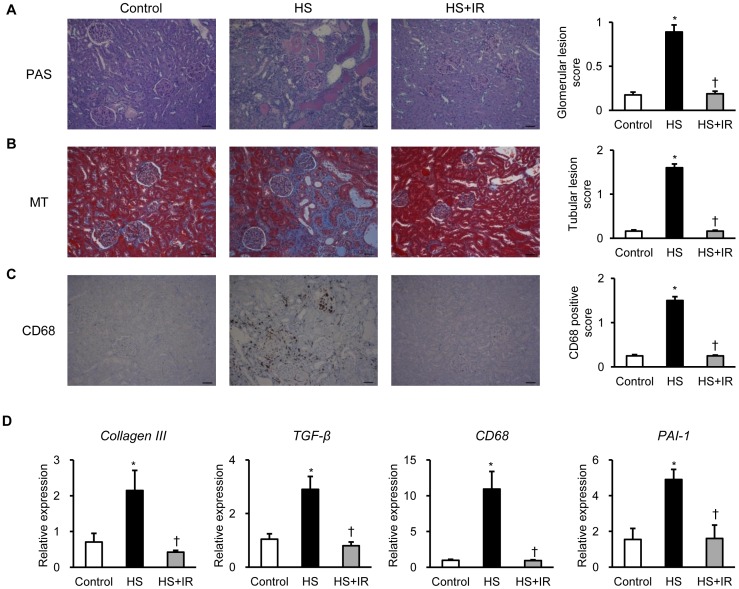
Effects of Dietary Iron Restriction on Renal Structure and Renal Gene Expression in Salt-Loaded SHRSP. Representative images of (A) PAS, (B) MT, and (C) CD68 staining of the kidney sections and quantitative analysis of (A) glomerular lesion score, (B) tubular lesion score, and (C) CD68 positive score in the Control (white bar), HS (black bar), and HS+IR (gray bar) groups (n = 6 in each group). Scale bars: 50 µm. (D) Renal gene expression of *Collagen III*, *TGF-β*, *CD68*, and *PAI-1* in the Control (white bar), HS (black bar), and HS+IR (gray bar) groups (n = 4–6 in each group). PAS, periodic acid-Schiff staining; MT, Masson’s trichrome staining. *p<0.05 versus the Control group, ^†^p<0.05 versus the HS group.

Next, we evaluated renal structure in salt-loaded SHRSP. Periodic acid-Schiff staining showed that HS diet induced severe glomerulosclerosis, tubular dilatation, and cast formation in the tubular lumen; however, these changes were suppressed in the HS+IR group (Figure 3A). In addition, Masson’s trichrome staining revealed that interstitial fibrosis was increased in the HS group, whereas it was markedly attenuated in the HS+IR group (Figure 3B). Furthermore, there was a significant increase in CD68 positive cells in the kidney of the HS group, while its expression was dramatically decreased in the HS+IR group (Figure 3C). Consistent with these findings, iron restriction suppressed the increased gene expression of collagen type III, TGF-β, CD68, and PAI-1 in the kidney of the HS group (Figure 3D).

**Figure 4:**
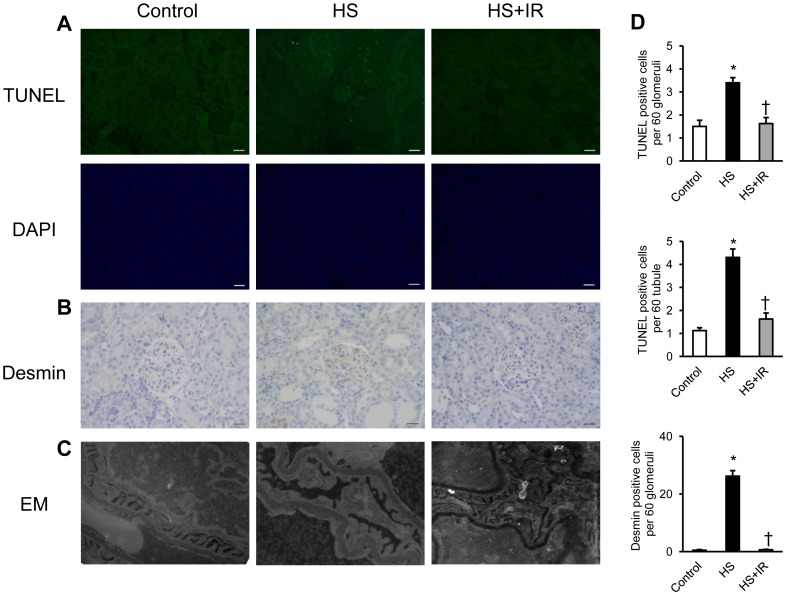
Effects of Dietary Iron Restriction on Renal Apoptosis and Podocyte Injury in Salt-Loaded SHRSP. Representative images of (A) TUNEL, DAPI, and (B) desmin staining of the kidney sections. Scale bars: 50 µm. (C) Representative images of electron microscopy of the kidney sections. (D) Quantitative analysis of TUNEL-positive cells per glomeruli and tubule, and desmin positive cells per glomeruli in the Control (white bar), HS (black bar), and HS+IR (gray bar) groups (n = 4–6 in each group). TUNEL, terminal deoxynucleotidyl transferase–mediated dUTP nick-end labeling staining; DAPI, 4′,6-diamidino-2-phenylindole staining; EM, electron microscopy. *p<0.05 versus the Control group, ^†^p<0.05 versus the HS group.

To further evaluate the renoprotective effects of iron restriction in salt-loaded SHRSP, we next investigated renal apoptosis and glomerular podocyte damage in these groups. HS diet induced an increase in TUNEL positive cells in the kidney, whereas it was markedly suppressed in the HS+IR group (Figure 4A,D). Moreover, increased expression of desmin, a marker of podocyte injury, was found in the kidney of the HS groups, while it was reduced in the HS+IR group (Figure 4B,D). Transmission electron microscopy showed that podocyte foot processes were effaced in the kidney of the HS group; however, it was attenuated in the HS+IR group (Figure 4C). Collectively, these data indicate that iron restriction attenuates the development of hypertensive nephropathy in salt-loaded SHRSP.

## Discussion

This study provides the first evidence to our knowledge that iron plays a role in the development of hypertensive nephropathy. Moreover, we show for the first time that dietary iron restriction attenuates the development of severe hypertension and nephrosclerosis, thereby improving survival rate in salt-loaded SHRSP. Interestingly, massive iron accumulation was observed in the tubules of salt-loaded SHRSP. In addition, renal iron content was increased in salt-loaded SHRSP. These results suggest a novel mechanism by which iron participates in the pathophysiology of hypertensive nephropathy and establish the effects of iron restriction on salt-induced nephrosclerosis.

Iron is a necessary mineral for life. However, it is involved in diverse pathological processes by catalyzing the formation of highly reactive oxidant. Thus, it is extremely important to consider the role of iron in the pathophysiology of various diseases. Indeed, iron accumulation is observed in the proximal tubules of human chronic renal disease [3] and rat models of renal diseases [4]–[6]. In addition, we have more recently reported that renal iron accumulation is increased in the tubules of CKD rats and that iron restriction attenuates the development of renal damage and hypertension in CKD rats [7]. In the present study, we investigated whether iron and intracellular iron transport proteins participated in the pathophysiology of hypertensive nephropathy and the effects of dietary iron restriction on nephrosclerosis in salt-loaded SHRSP. High salt intake worsens not only hypertension but also several cardiovascular diseases, such as hypertensive nephropathy [11]. In fact, salt loading on SHRSP leads to severe hypertension and hypertensive nephropathy [12]. However, how high salt intake exacerbates hypertensive nephropathy remains obscure. Furthermore, it is unknown whether iron is involved in the development of hypertensive nephropathy. Of note, we found massive iron accumulation in the tubules of salt-loaded SHRSP. Moreover, we demonstrated that iron restriction attenuated the development of hypertension, nephrosclerosis, thereby improving survival rate in salt-loaded SHRSP. Taken together, these findings suggest that iron plays a role in the development of hypertensive nephropathy.

Excess iron accumulation promotes increased oxidative stress, which leads to cell and tissue damage. To our best knowledge, this is the first report to show that renal iron content is increased in salt-loaded SHRSP. In fact, urinary 8-OHdG excretion was increased in the HS group, whereas dietary iron restriction reduced the increment of urinary 8-OHdG excretion under HS diet. Collectively, these results suggest that attenuation of oxidative stress may partially contribute to the renoprotective effects of iron restriction. Patients with CKD are prone to develop iron deficiency, and iron deficiency can be corrected by iron supplementation in patients with CKD. However, long-term concerns of iron supplementation in patients with CKD are increasing oxidative stress, worsening proteinuria, and tubular toxicity [13]. In this regards, we suggest from our results that iron deficiency may be induced as a negative feedback mechanism to protect against the iron-dependent renal damage in patients with CKD.

In the current study, high salt diet seems to induce massive iron accumulation in the tubules of SHRSP. This is the first demonstration of massive changes in renal iron metabolism due to salt loading. To investigate whether iron transporter is associated with the mechanism of salt-induced nephrosclerosis, we assessed cellular iron transport proteins, TfR1 and DMT-1, in the kidney of these rats. Of interest, we found increased protein expression of TfR1 and DMT-1 in the tubules of both HS and HS+IR groups, suggesting that renal iron reabsorption may occur in both HS and HS+IR group. Most cells regulate iron uptake by modulating the amount of TfR1. Low iron conditions normally lead to upregulate TfR1 expression. Conversely, high iron conditions induce to downregulate TfR1 expression [14]. Interestingly, renal TfR1 protein expression was normally increased in the HS+IR group under iron restricted conditions, while TfR1 protein expression was increased in the HS kidney without artificial iron restricted conditions, suggesting that dysregulation of cellular iron transport proteins may occur in the kidney of salt-loaded SHRSP. Further studies are necessary for exploring the mechanisms by which high salt diet induces massive iron accumulation in the tubules of SHRSP.

In conclusion, iron is involved in the development of hypertensive nephropathy. Iron restriction attenuates the development of severe hypertension and nephrosclerosis, thereby improving survival rate in salt-loaded SHRSP. These effects seem to be mediated by inhibition of oxidative stress. Understanding the effects of iron restriction on salt-induced nephrosclerosis may contribute to the development of a novel therapeutic strategy for hypertensive nephropathy.
